# A phenomenological model for cell and nucleus deformation during cancer metastasis

**DOI:** 10.1007/s10237-018-1036-5

**Published:** 2018-05-29

**Authors:** Jiao Chen, Daphne Weihs, Marcel Van Dijk, Fred J. Vermolen

**Affiliations:** 1 Delft Institute of Applied Mathematics, Delft University of Technology, Delft, The Netherlands; 2 Faculty of Biomedical Engineering, Technion-Israel Institute of Technology, 3200003 Haifa, Israel

**Keywords:** Cell deformation, Nucleus deformation, Monte Carlo simulations, Cancer metastasis, Cell-based model

## Abstract

Cell migration plays an essential role in cancer metastasis. In cancer invasion through confined spaces, cells must undergo extensive deformation, which is a capability related to their metastatic potentials. Here, we simulate the deformation of the cell and nucleus during invasion through a dense, physiological microenvironment by developing a phenomenological computational model. In our work, cells are attracted by a generic emitting source (e.g., a chemokine or stiffness signal), which is treated by using Green’s Fundamental solutions. We use an IMEX integration method where the linear parts and the nonlinear parts are treated by using an Euler backward scheme and an Euler forward method, respectively. We develop the numerical model for an obstacle-induced deformation in 2D or/and 3D. Considering the uncertainty in cell mobility, stochastic processes are incorporated and uncertainties in the input variables are evaluated using Monte Carlo simulations. This quantitative study aims at estimating the likelihood for invasion and the length of the time interval in which the cell invades the tissue through an obstacle. Subsequently, the two-dimensional cell deformation model is applied to simplified cancer metastasis processes to serve as a model for in vivo or in vitro biomedical experiments.

**Electronic supplementary material:**

The online version of this article (10.1007/s10237-018-1036-5) contains supplementary material, which is available to authorized users.

## Introduction

Cell locomotion is closely involved in various physiological and pathological processes. For example, migration of leukocytes is important for the inflammatory response and movement of fibroblasts and also vascular endothelial cells are essential for wound healing (Lauffenburger and Horwitz 1996). On the contrary, cell migration can play a detrimental role during cancer metastasis, where the dissemination of cancer cells initializes the invasion-metastasis cascade as introduced by Chambers et al. (2002b), Fidler (2003), Lambert et al. (2017).

The diversity of cancers exceeds 200 distinct disease entities, which have differences in the normal cells of origin and similarities in subsequent cancer metastasis. Compared to primary tumors, metastatic cancers cause the overwhelming majority of cancer-associated deaths as high as 90% (Lambert et al. 2017; Seyfried and Huysentruyt 2013; Gupta and Massagué 2006). During the metastatic spreading of tumors, cancer cells can undergo transitions between two forms of movement, which are the amoeboid mode and the mesenchymal mode to optimize their invasiveness (Paňková et al. 2010; Sahai and Marshall 2003). Moreover, Pinner and Sahai (2008) observe that cancer cells are able to move quickly (up to 15 $\upmu \mathrm{m/min}$) like some leukocytes and rapidly change their shapes and directions of migration in an amoeboid manner with intravital confocal microscopy technology. Amoeboid movement could happen in the absence of matrix protease (Wolf et al. 2003; Wyckoff et al. 2006) where cancer cells alternatively generate large contractile force pushing fibers of matrix away and squeeze between small paths. However, if the contractile force is insufficient to deform the stiff extracellular matrix (ECM), the matrix metallo-proteases (MMP’s) will be secreted by cancer cells to degrade the ECM and thereby invade further (Kalebic et al. 1983; Wolf et al. 2013). In summary, cancer cells frequently chemically and/or mechanically ‘dig’ their ways through ECM in order to reach the distinct parts of the body.

When a single cancer cell is metastasizing through a narrow cavity, it must deform its morphology by extending its membrane into an elongated protrusion; this is often driven by external signals such as chemotaxis, durotaxis or tensotaxis. Large cell deformations will also induce changes in the nucleus morphology. Extensive deformation of the nucleus can induce damage, and reduce the nuclear envelope integrity, see for instance the work by Denais et al. (2016). However, the cancer cell is also capable of repairing its ruptured nuclear envelope and damaged DNA after the penetration. Then, the cell may be able to further promote cancer development. Thus, as noted by Denais et al. (2016), the stage of nuclear envelop rupture could represent a particularly fragile point, thereby providing an opportunity to develop new anti-metastatic cancer drugs to inhibit DNA repair and increase cell death. Cell deformation during cancer metastasis has been difficult to study in detail both in vivo and vitro, and further understanding of cell deformation mechanisms is crucially important. In cases where the pore sizes are much smaller than the size of the nucleus, the nucleus mostly arrests and fails to penetrate the pore due to a defective nuclear deformability. On the contrary, with pore diameters above a threshold, e.g., 7 $\upmu \mathrm{m}$ in the work (Wolf et al. 2013), MMP-independent migration in dense ECM relies on the hourglass-shaped deformation of the nucleus. Hence, in our current work, we develop a mathematical model to investigate the correlation between the deformation of a cell and of its nucleus, and show the dynamic changes in cell mechanostructure that occur during the invasion process.

Mathematical modeling has been shown to be an important tool to quantify the relations in many biomedical processes such as wound healing, cell migration and tumor progression in various scales. Cell deformation and migration models exist in the colony scale, e.g., in the works by Rey and Garcia-Aznar (2013), Byrne and Drasdo (2009), and Vermolen and Gefen (2012), where the cell geometry is fixed to be circular or spherical, respectively, in two- and three-dimensional simulations. On a smaller scale, one looks at the deformation of individual cells, and to this extent, cellular automata models have been developed and combined with finite-element strategies by Borau et al. (2014) and Oers et al. (2014). Other cell deformation models are based on phase-field models, like in the work by Marth and Voigt (2014), or on viscoelasticity with moving meshes as in (Madzvamuse and George 2013). A phenomenological approach to cell migration and deformation is proposed in Vermolen and Gefen (2013) and Vermolen et al. (2014), wherein the latter work cell migration and deformation have been modeled in relation with the immune response system where white blood cells migrate out of the venules and transmigrate through the venule walls to chase and engulf pathogens. Moreover, Odenthal et al. (2013) introduce a deformable cell model to describe the mechanical communication among the interacting cells and between the cell and its environment. Another deformable model regarding the interactions with emphasis on the relationship between varying matrix geometries and adhesion, contractility as well as cell velocity can be found in (Tozluoğlu et al. 2013). In terms of the nucleus deformable models, Moussavi-Baygi et al. (2011) establish a coarse-grained model of the nuclear pore complex to simulate the nucleocytoplasmic transport. As the increasing attention in the cell mechanics, agent-based models are booming, see (van Liedekerke et al. 2015), where three types agent-based models are described.

Cao et al. (2016) develop a chemomechanical model to investigate the impacts of transmigration through confined interstitial spaces on the geometrical and mechanical features of the cell nuclei. In their model, the shape alterations of the cell and nucleus during the transendothelial migration driven by actomyosin contraction force can perturb genomic organization, which in turn affects the behavior of the cells. More nuclear profiles regarding chromatin deformations and nuclear envelope deformations during transmigration are further investigated. This mechanical model successfully predicts the morphological evolution when one cell transmigrate an endothelial gap (Cao et al. 2016). In comparison, our model extends the process and behavior of cell transmigration driven by a chemical/stiffness signal during cancer metastasis, whereas the most inner cellular mechanical properties are neglected for sake of simplicity.

None of the aforementioned studies, however, have taken into account the Monte Carlo uncertainty quantification in the cell deformation modeling. Our work aims at modeling the interaction between cell deformation (due to migration) and the deformation of the nucleus as well as quantitative analysis of unknown parameters by Monte Carlo simulations. We quantify the correlation between nuclear deformation relaxation and the cell’s ability to penetration through narrow passages, which is important in the context of metastatic invasion. Section 2 describes the mathematical model in terms of the equations, subsequently, the numerical method is presented in Sect. 3, which is followed by the description of the results in Sect. 4. Finally, conclusions are drawn in Sect. 5.

## The mathematical model

This section introduces the model in terms of the mathematical relations. We start with the deformation of cell and its nucleus in two dimensions and extend the formalism to three spatial dimensions subsequently. Moreover, the model is applied to simplified physiological transmigration of cancer cells and six parameters are studied by Monte Carlo simulations.

### The model in two dimensions

**Table 1:** Comparison of CPU time and the cell penetration time $\tau$

*N*	10	30	50	100
CPU time (s)	2.43	5.07	7.81	14.85
$\tau$ (h)	0.3771	0.3735	0.3812	0.3906

**Fig. 1:**
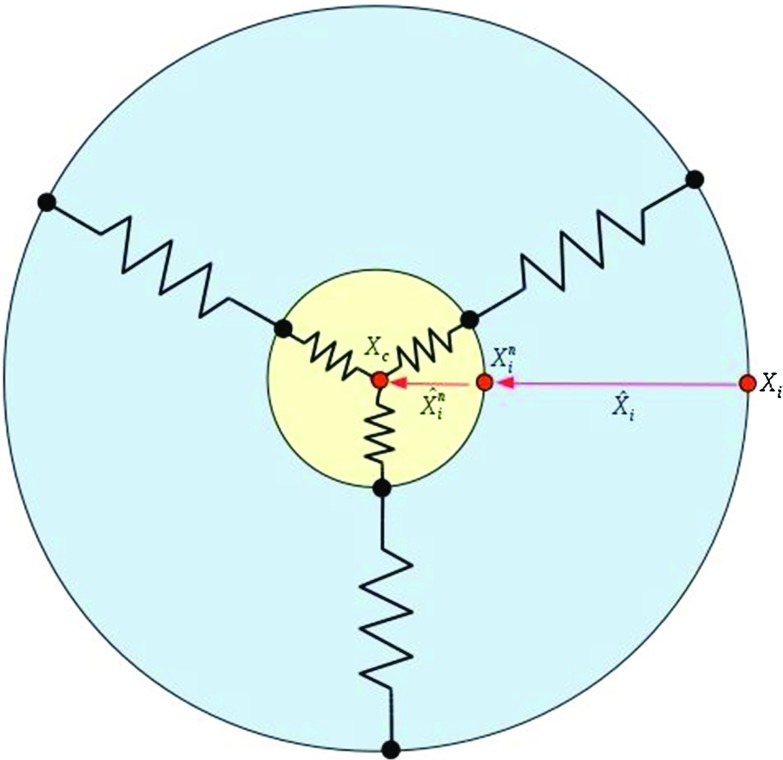
A schematic of the distribution of the nodal points on the cell boundary membrane and the surface of the nucleus. The cytoskeleton is represented as a collection of springs. The red dots, $\mathbf{x}_i$, $\mathbf{x}_i^n$ and $\mathbf{x}_c$, denote nodal points on the cell membrane, nucleus surface and x coordinate of the cell center of mass, respectively. The vectors $\hat{\mathbf{x}}_i$ and $\hat{\mathbf{x}}_i^n$ are represented in red arrows

The nucleus must move in coordination with the cell cytoskeletal dynamics at the front edge and rear end (Friedl et al. 2011). To mimic this cell’s cytoskeleton, a cell is treated as a collection of 30 parallel nodal points that are located on the cell membrane and on the outer boundary of the cell nucleus. We have compared the number of nodal points *N* ($N = 10, 30, 50, 100$) and we found that if the cell is freely moving that the pattern is hardly influenced by the number of springs, whereas the CPU time increases proportionally with the number of springs. If the number of springs is very large, then the time step needs to be adjusted if the cell is in contact with an obstacle. In particular, it may happen if the resolution is too high that the nodal points on the cell boundary overtake each other when they are in (partial) contact with a rigid boundary. Taking the model in Fig. 6 as an example (no perturbation of the random walk), the CPU time and penetration time $\tau$ are compared with various *N* in Table 1. The table shows that CPU time increases, whereas the cell penetration time $\tau$ is comparable with the increase of *N*. Each node on the cell membrane is connected to its corresponding node on the surface of the cell nucleus. On each of the nodes on the cell membrane surface, an external signal, such as a concentration gradient in the case of chemotaxis or durotaxis, is computed. This signal determines the movement of the nodal point. Next to this signal, the migration of the nodal point is determined by its position relative to its corresponding point of the nucleus boundary via the deformation relaxation of the cell’s cytoskeleton. In this way, the deformation and migration of the cell is modeled and sketched in Fig. 1.

We consider a generic signal, of which the gradient determines the migration of the nodal points on the cell boundary membrane. This signal could be the extracellular stiffness or the concentration of a chemoattractant or a light intensity for instance. In the work by Massalha and Weihs (2017), the gel-stiffness-dependent differences among cells with various metastatic potentials have been observed to be correlated with cancer invasiveness, where the metastatic cells apply a wide spectrum of traction forces (100–600 nN) for their adhesion to a stiffer gel. For the sake of presentation, we denote the intensity of the signal by $c(t,\mathbf{x})$, where *t* and $\mathbf{x}$, respectively, denote time and spatial position. The signal, as well as its gradient, can be obtained from a given relationship in which the gradient is determined either analytically or numerically. A numerical evaluation in a finite-element framework could be carried out by for instance gradient recovery techniques or by mixed finite-element formulations. In the present paper, we consider a chemical attractant, such as a generic growth factor that attracts the cells. For the sake of illustration, we consider a point source since this allows for a simple treatment using Green’s Fundamental solutions. To this extent, let the emitting source of the chemoattractant by positioned on $\mathbf{x}_S$, then on an unbounded domain, we solve

1 $$\begin{aligned} -D \Delta c = \gamma _S \delta (\mathbf{x} (t)- \mathbf{x}_S(t)). \end{aligned}$$

Here, *D* and $\gamma _S$ represent the diffusion coefficient of the chemokine and the secretion rate of the source. Moreover, $\delta$ is the Delta Dirac function, while $\mathbf{x} (t)$ and $\mathbf{x}_S(t)$ denote positions of the nodal points on the cell membrane and the source. The fundamental solution to this equation in an unbounded domain is used in two spatial dimensions as follows,

2 $$\begin{aligned} c(t, \mathbf{x}) = -\frac{\gamma _S}{2\pi D}\ln (\mathbf{x}(t)-\mathbf{x}_S(t)). \end{aligned}$$

In the presence of multiple cells, the superposition principle is used to construct the solution. We note that the signal can be taken as generic as one wishes. The above equation just serves as an illustration. Note that the above equation predicts negative values for the concentration if the distance between the point of observation and the source is too large. In our simulations, the distances are such that the above expression predicts nonnegative values only. Let the set $\mathbf{x}_i(t)$ and $\mathbf{x}^n_i(t)$, respectively, denote the nodal points on the cell boundary membrane and on the surface of the nucleus of the cell. Then, the migration of the nodal points on the cell boundary membrane is determined by

3 $$\begin{aligned} \begin{aligned} \mathrm{d}{} \mathbf{x}_i(t)= \,&\beta \nabla c(t,\mathbf{x_i}(t)) \mathrm{d}t + \alpha \left( \mathbf{x}_i^n(t) + \hat{\mathbf{x}}_i - \mathbf{x}_i(t) \right) \mathrm{d}t\\&+\,\eta \mathrm{d}{} \mathbf{W}(t), \quad i \in \{1,\ldots ,N\}. \end{aligned} \end{aligned}$$

Here $\hat{\mathbf{x}}_i$ represents the vector connecting the initial position of nodal point *i* on the cell boundary membrane to the initial position of point *i* on the cell nucleus (see Fig. 1). This vector defines the equilibrium cell shape. In this text, we only consider circular and spherical cells, however, this formulation allows to consider cells of generic shapes such as dendritic shapes. Furthermore, $\beta$ stands for the cell’s response to external signals, and $\alpha > 0$ denotes the cell’s deformation relaxation. Over a spectrum of cell types, the mobility of the cell boundary has a locally persistent random character (Lauffenburger and Horwitz 1996), thus the last term takes care of the randomness movements of each node, where $\eta$ is a constant and $\mathrm{d}{} \mathbf{W}(t)$ denotes a vector Wiener process with independent samples from a normal distribution with zero mean and variance $\mathrm{d}t$. The above equation warrants convergence to the equilibrium cell shape if there is no external stimulus for the deformation and migration of the cell.

Next, we introduce the equation of motion for the nodal points on the surface of the nucleus. We proceed similarly to the previous treatment of the nodal points on the cell boundary membrane, where we link the positions of the nodal points on the surface of the nucleus to their counterparts on the boundary membrane as well as to the position of the midpoint of the cell nucleus. To this extent, we obtain for $i \in \{1,\ldots ,N\}$

4 $$\begin{aligned} \begin{aligned} \mathrm{d}{} \mathbf{x}_i^n(t) =\,&\alpha ^n \left( \mathbf{x}_c(t) + \hat{\mathbf{x}}_i^n - \mathbf{x}_i^n(t) \right) \mathrm{d}t - \\&\alpha \left( \mathbf{x}_i^n(t) + \hat{\mathbf{x}}_i - \mathbf{x}_i(t) \right) \mathrm{d}t +\eta \mathrm{d}{} \mathbf{W}(t). \end{aligned} \end{aligned}$$

Here $\alpha ^n$, $\mathbf{x}_c$ and $\hat{\mathbf{x}}_i^n$, respectively, stand for the deformation relaxation of the nucleus, the position of the center of the nucleus and the vector connecting the initial position of point *i* on the surface of the nucleus to the initial center of the nucleus (see Fig. 1). Furthermore, the random character of the mobility of the boundary of the nucleus has been taken into account. This treatment of the points on the surface of the nucleus provides the interaction between the nucleus and the cell membrane. However, this interaction such that the deformation of the nucleus is delayed and damped with respect to the deformation of the membrane.

In order to maintain the right orientation of the cell, we also introduce the rotation matrix after rotation of an angle $\phi$ relative to the *x*-axis:

5 $$\begin{aligned} B(\phi ) = \begin{pmatrix} \cos (\phi ) &{} -\sin (\phi ) \\ \sin (\phi ) &{} \cos (\phi ) \end{pmatrix}, \end{aligned}$$

which transforms a vector $\mathbf{x} \in \mathbb {R}^2$ to

6 $$\begin{aligned} \mathbf{x} \longrightarrow B(\phi ) \mathbf{x}. \end{aligned}$$

**Fig. 2:**
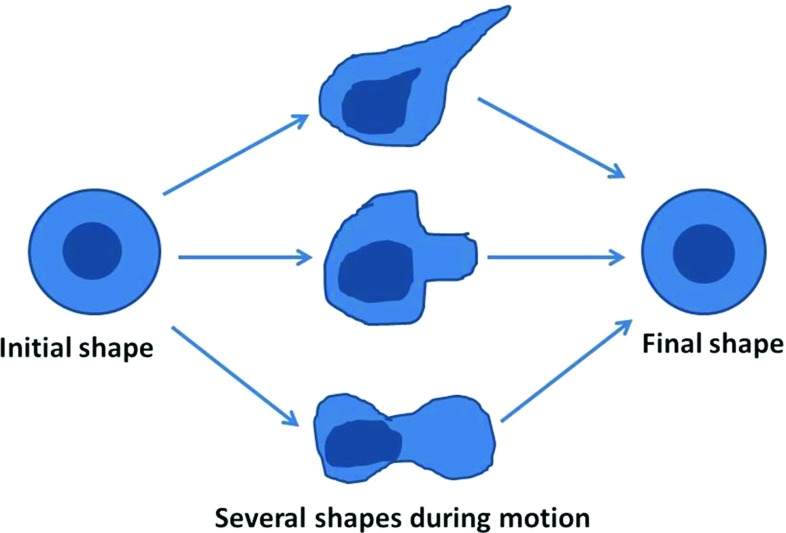
An example of movement and polarity of the cell

The rotation matrix $B(\phi )$ is used to determine the new equilibrium points of the cell boundary membrane and of the surface of the nucleus. Therefore, one cell is able to converge to its initial shape as well as to its rotation as a result of its migration to simulate the cell morphological polarization, see Fig. 2 for a sketch. The angle $\phi$ is determined such that it is closest to the current position of all the nodal points on the cell boundary membrane:

7 $$\begin{aligned} \phi = \mathop {{{\mathrm{arg\,min}}}}\limits _{\phi \in [0,2\pi )} \left( \sum _{i=1}^N || B({\tilde{\phi }}) \tilde{\mathbf{x}}_i - \mathbf{x}_i(t) ||^2 \right) , \end{aligned}$$

where $\tilde{\mathbf{x}}_i$ represents the initial position of the i-th node on the cell membrane surface with the cell center position at time *t*. After the above problem has been solved, then the angle of rotation of the cell with respect to the x-axis is known. This angle, $\phi$, is substituted into the equations of motion for all nodal points on the cell membrane surface and on the surface of the cell nucleus, which gives for $i \in \{1,\ldots ,N\}$:

8 $$\begin{aligned} \begin{aligned} \mathrm{d}{} \mathbf{x}_i(t) =\,&\beta \nabla c(t,\mathbf{x_i(t)}) \mathrm{d}t \\&+\alpha \left( \mathbf{x}_i^n(t) + B(\phi ) \hat{\mathbf{x}}_i - \mathbf{x}_i(t) \right) \mathrm{d}t +\eta \mathrm{d}{} \mathbf{W}(t), \end{aligned} \end{aligned}$$

and

9 $$\begin{aligned} \begin{aligned} \mathrm{d}{} \mathbf{x}_i^n(t) =\,&\alpha ^n \left( \mathbf{x}_c(t) + B(\phi ) \hat{\mathbf{x}}_i^n - \mathbf{x}_i^n(t) \right) \mathrm{d}t - \\&\alpha \left( \mathbf{x}_i^n(t) + B(\phi ) \hat{\mathbf{x}}_i - \mathbf{x}_i(t) \right) \mathrm{d}t +\eta \mathrm{d}{} \mathbf{W}(t). \end{aligned} \end{aligned}$$

Next we consider the treatment of an obstacle. Imagine that the surface or contour (in 2D) of the obstacle is given by $\partial \Omega$ and let the unit normal vector be given by $\mathbf{n}$, then we require that the component of the migration vector, $\mathrm{d} \mathbf{x}_i(t)$ has no component in the normal direction of the obstacle’s surface, hence we require that the inner product of $\mathrm{d} \mathbf{x}_i(t)$ and $\mathbf{n}$ vanishes, that is

10 $$\begin{aligned} \left( \mathrm{d} \mathbf{x}_i(t),\mathbf{n}(\mathbf{x}_i(t))\right) = 0, \qquad \text {if } \quad \mathbf{x}_i(t) \in \partial \Omega . \end{aligned}$$

From this we subtract the component of $d \mathbf{x}_i(t)$ in the direction of $\mathbf{n}$, hence this gives the following adjustment

11 $$\begin{aligned} \begin{aligned}&\mathrm{d} \mathbf{x}_i(t) \longleftarrow \mathrm{d} \mathbf{x}_i(t) - \left( \mathrm{d} \mathbf{x}_i(t),\mathbf{n}(\mathbf{x}_i(t))\right) \mathbf{n}(\mathbf{x}_i(t)), \\&\text {if } \quad \mathbf{x}_i(t) \in \partial \Omega . \end{aligned} \end{aligned}$$

Note that herewith the obstacle slows down the migration of the cells. This principle is also applied if cells are colliding into each other. The current model simplifies the mechanics of the cell considerably. Inertial effects would change equations (3, 4, 8, 9) into second order equations with respect to the time derivative. The first term with the first-order time derivative is generally associated with friction or damping. Since in most studies inertia is neglected compared to friction terms (Galle et al. 2005; Drasdo et al. 1995; Odell et al. 1980), we are faced with a system of first-order differential equations. Note that the incorporation of more complex mechanics also increases the parameter space of the model, where input parameters are often hard to get.

### Extension to three spatial dimensions

Chemotaxis migration is modeled by using the Green’s function as a solution of Eq. (1). However, compared to the 2D model, the Green’s functions in 3D changes to,

12 $$\begin{aligned} c(t,\mathbf{x})= \frac{\gamma _S}{4\pi D \Vert \mathbf{x}(t)-\mathbf{x}_s(t)\Vert ^2}, \end{aligned}$$

where both of $\mathbf{x (t)}$ and $\mathbf{x}_s(t)$ have *x*, *y* and *z* components.

The surface of the outer membrane and the nuclear surface are divided into mesh points. For this case, superpositions of the three-dimensional Green’s Fundamental solutions are used, as well as the same principles for collision with obstacles and other cells. Further, the rotation can be imposed around all the three coordinate axes, and to this extent, the rotation matrix $B(\phi )$, entailing a rotation about the *z*-axis, where $\phi$ denotes the angle with respect to the *x*-axis, we extent the rotation matrix to all three coordinate axes:

13 $$\begin{aligned} B(\phi _x,\phi _y,\phi _z) = B_x(\phi _x) \cdot B_y(\phi _y) \cdot B_z(\phi _z). \end{aligned}$$

Here $B_q(\phi _q)$ denotes the rotation matrix about the *q*–axis ($q \in \{x,y,z\}$), given by

14 $$\begin{aligned} \begin{array}{l} B_x(\phi _x) = \begin{pmatrix} 1 &{} 0 &{} 0 \\ 0 &{} \cos (\phi _x) &{} -\sin (\phi _x) \\ 0 &{} \sin (\phi _x) &{} \cos (\phi _x) \end{pmatrix}, \\ \\ B_y(\phi _y) = \begin{pmatrix} \cos (\phi _y) &{} 0 &{} -\sin (\phi _y) \\ 0 &{} 1 &{} 0 \\ \sin (\phi _y) &{} 0 &{} \cos (\phi _y) \end{pmatrix}, \\ \\ B_z(\phi _z) = \begin{pmatrix} \cos (\phi _z) &{} -\sin (\phi _z) &{} 0\\ \sin (\phi _z) &{} \cos (\phi _z) &{} 0 \\ 0 &{} 0 &{} 1 \end{pmatrix}. \end{array} \end{aligned}$$

All other principles remain the same and rotation is determined using a minimization with respect to the three coordinate angles.

### The application to cancer metastasis

The ECM has preexisting pores (diameter varies from 1 to 20 $\upmu \mathrm{m}$) or fiber-like (ranging from less than 3–30 $\upmu \mathrm{m}$ in width) and channel-like (varying from 100 to 600 $\upmu \mathrm{m}$) tracks (Paul et al. 2017). Furthermore, cells are viscoelastic objects such that morphological deformation happens frequently during the cancer invasion process (Mak and Erickson 2013). Thence, our model for cell and nucleus deformation is applied to a simplified process occurring during cancer metastasis in a pore and a channel-like microenvironment. During the invasion, a cancer cell is normally able to squeeze obstacles like cells, tissue, capillary-sized vessels and deform itself as well as its nucleus to penetrate and seed in other organs. In the model, we use a constraint cavity with varying roughnesses to simulate the microenvironment during cancer spread, which is mimicked using a trigonometric function as follows.

15 $$\begin{aligned} y = \pm (y_0 + \epsilon \sin (\omega x)), \end{aligned}$$

where *y* depicts the rough tube bounds and $y_0$ is a constant used to adjust the width of a tube. Through changing $\epsilon$ and $\omega$, different roughnesses (varying amplitudes and frequencies) can be simulated.

## The numerical method

### Time integration

We describe the two-dimensional case and provide information for the 3D-case if it substantially differs from the 2D-case. Initially the cell outer membrane surface is divided into *N* mesh points with respect to the cell center located on $(x_c,y_c)$ as follows

16 $$\begin{aligned} \begin{aligned} \mathbf{x}_i(0) =&\left( x_c + R \cos \left( 2\pi \frac{(i-1)}{N}\right) ,\right. \\&\left. y_c + R \sin \left( 2\pi \frac{(i-1)}{N}\right) \right) , \\&\quad i \in \{1,\ldots ,N\}. \end{aligned} \end{aligned}$$

where we assume the cell to be circular in 2D with radius *R*. The counterparts of the mesh points on the nuclear surface are given by

17 $$\begin{aligned} \begin{aligned} \mathbf{x}^n_i(0) =&\left( x_c + R^n \cos \left( 2\pi \frac{(i-1)}{N}\right) ,\right. \\&\left. y_c + R^n \sin \left( 2\pi \frac{(i-1)}{N}\right) \right) ,\\&\quad i \in \{1,\ldots ,N\}, \end{aligned} \end{aligned}$$

where $R^n < R$ represents the radius of the cell nucleus. The 3D spherical cell is described analogously for $i \in \{1,\ldots ,N\}$ and $j \in \{1,\ldots ,M\}$

18 $$\begin{aligned} \begin{aligned} \mathbf{x}_{i,j}(0) =&\left( x_c + R \cos \left( 2\pi \frac{(i-1)}{N}\right) \sin \left( \pi \frac{(j-1)}{M}\right) ,\right. \\&y_c + R \sin \left( 2\pi \frac{(i-1)}{N}\right) \sin \left( \pi \frac{(j-1)}{M}\right) ,\\&\left. z_c +R \cos \left( \pi \frac{(i-1)}{M}\right) \right) , \end{aligned} \end{aligned}$$

and on the nuclear surface by

19 $$\begin{aligned} \begin{aligned} \mathbf{x}_{i,j}(0) =&\left( x_c + R^n \cos \left( 2\pi \frac{(i-1)}{N}\right) \sin \left( \pi \frac{(j-1)}{M}\right) ,\right. \\&y_c + R^n \sin \left( 2\pi \frac{(i-1)}{N}\right) \sin \left( \pi \frac{(j-1)}{M}\right) ,\\&z_c +R^n \cos \left( \pi \frac{(i-1)}{M})\right) . \end{aligned} \end{aligned}$$

These initial values can be applied to the multi-cell configuration similarly. To determine the positions of the nodal points on the outer membrane surface, we use an IMplicit-EXplicit (IMEX) time integration to update the positions at the next time step in such a way that the linear parts are treated in an Euler backward method, whereas the nonlinear parts are treated using a forward Euler method. This treatment has been chosen to avoid the need of solving a nonlinear system using an iterative procedure. This treatment results into the following equation (for the single-cell two-dimensional case) for the nodes on the outer membrane

20 $$\begin{aligned} \begin{aligned} \mathbf{x}_i(t^{p+1}) =\,&\mathbf{x}_i(t^p)+ \Delta t \cdot ( \beta \nabla c_i(t^{p+1}) +\alpha ( \mathbf{x}_i^n(t^{p}) \\&+\, \hat{\mathbf{x}}_i - \mathbf{x}_i(t^{p+1}))) +\eta \Delta \mathbf{W}, \end{aligned} \end{aligned}$$

and

21 $$\begin{aligned} \begin{aligned} \mathbf{x}_i^n(t^{p+1}) = \,&\mathbf{x}_i^n(t^p)+ \Delta t \cdot ( - \alpha ( \mathbf{x}_i^n(t^{p}) + \hat{\mathbf{x}}_i - \mathbf{x}_i(t^{p+1})) \\&+ \alpha ^n ( \mathbf{x}_c(t^{p}) + \hat{\mathbf{x}}_i^n - \mathbf{x}^n_i(t^{p+1})))+\eta \Delta \mathbf{W}, \end{aligned} \end{aligned}$$

for the nodes on the nuclear surface, for $i \in \{1,\dots ,N\}$. Here, $\Delta \mathbf{W}$ is a two-dimensional Wiener process with variables from a normal distribution with zero mean and $\Delta t$ variance. For the definition and introduction of the vector Wiener process, one can refer to Steele (2012). For the gradient of the concentration (or any other signal that triggers cell migration and deformation), we use the following IMEX convention based on the Green’s Fundamental solutions in 2D (in 3D analogously)

22 $$\begin{aligned} \nabla c_i^{p+1} = \frac{\gamma _S(t^{p+1}) (\mathbf{x}_S(t^{p+1}) - \mathbf{x}_i(t^{p+1}))}{\pi D || \mathbf{x}_S(t^{p}) - \mathbf{x}_i(t^{p}) ||^2}. \end{aligned}$$

### Cell shape

In order to compute the coordinate of the cell center of mass, we need the area or volume of the cell and nucleus. The area *A*(*t*) in 2D is computed by realizing that the cell is a polygon, which follows from

23 $$\begin{aligned} \begin{aligned} A(t)&= \int _{\partial \Omega } x(t) n_x(t) d \Gamma \\&\approx \frac{1}{2}\left[ \sum _{i \in \{1,\dots , N-1\}} (x_{i+1} + x_i) (y_{i+1}-y_i) + \right. \\&\left. (x_{1} + x_N)) (y_{1}-y_N) \right] . \end{aligned} \end{aligned}$$

For the 3D counterpart, we divide the cell into triangles (in order to allow any finite-element surface mesh), and compute the volume *V*(*t*) of the cell by

24 $$\begin{aligned} \begin{aligned} V(t)&= \int _{\partial \Omega } x(t) n_x(t) \mathrm{d} S \\&= \sum _{j\in \{1,\ldots ,N_{\mathrm{el}}\}} \int _{\partial \Omega _j} x(t) n_x(t) \mathrm{d} S \\&\approx \sum _{j \in \{1,\ldots ,N_{\mathrm{el}}\}} \frac{|\Delta _j| n_x}{6} \cdot \sum _{m \in \{j_1,j_2,j_3\}} x_m, \end{aligned} \end{aligned}$$

where $N_{\mathrm{el}}$ denotes the number of triangles that are used to approximate the cell (or nuclear) surface, and $j_1,~j_2$ and $j_3$ refer to the indexes of the vertices of triangle *j*. Further, $\frac{1}{2} |\Delta _j|$ denotes the area of the *j*-th triangle, where we compute $\Delta _j$ by

25 $$\begin{aligned} |\Delta _j| = || ( \mathbf{x}_{j2}-\mathbf{x}_{j1} ) \times ( \mathbf{x}_{j3} - \mathbf{x}_{j1} ) ||, \end{aligned}$$

and the unit outward normal vector by

26 $$\begin{aligned} \mathbf{n}_j = \frac{(\mathbf{x}_{j2}-\mathbf{x}_{j1} ) \times ( \mathbf{x}_{j3} - \mathbf{x}_{j1} ) }{|| ( \mathbf{x}_{j2}-\mathbf{x}_{j1} ) \times ( \mathbf{x}_{j3} - \mathbf{x}_{j1} ) ||}, \end{aligned}$$

and hence to compute $|\Delta _j| n_x$, it suffices to take the x–coordinate of $(\mathbf{x}_{j2}-\mathbf{x}_{j1} ) \times ( \mathbf{x}_{j3} - \mathbf{x}_{j1} )$.

For the 3D-case, we note that the area is computed by summing the areas of all the triangles, that is

27 $$\begin{aligned} A_b(t) \approx \frac{1}{2} \sum _{j \in \{1,\ldots ,N_{\mathrm{el}}\}} || (\mathbf{x}_{j2}-\mathbf{x}_{j1}) \times (\mathbf{x}_{j3}-\mathbf{x}_{j1}) ||. \end{aligned}$$

### The Monte Carlo simulations

In our model, most experimental data is difficult or even impossible to collect, therefore, we refer to other literature data or estimate the input data and thereby evaluating the quantification of the propagation of uncertainty in the variables is very important. To investigate the output influence and correlation among variables, Monte Carlo simulations are carried out based on the model of cancer metastasis. There, a cell transmigrates through a narrow rough tubular path to get from one part of the surrounding tissue to another part. Passage through the tube requires deformation of the cells’ cytoplasm and nucleus and affects the corresponding penetration time $\tau$ which is quantified under different conditions.

Suppose the variable $X \in \{D, \beta , \alpha , \alpha ^n\}$ follows a normal distribution $X \sim N(\mu , \sigma ^2)$, where $\mu$ and $\sigma$ represent the mean of the distribution and the standard deviation. Then, the stochastic variable *X* could be generated by

28 $$\begin{aligned} X = (randn(N_\mathrm{s},1)\times \sigma ) + \mu , \end{aligned}$$

here $N_\mathrm{s}$ denotes the number of samples. The strength of the linear association between every variable and penetration time $\tau$ is quantified by the correlation coefficient *r* given by

29 $$\begin{aligned} r =\frac{\sum \limits _{j=1}^N(X_j-\bar{X})(\tau _j-\bar{\tau })}{\left[ \sum \limits _{j=1}^N(X_j-\bar{X})^2\sum \limits _{j=1}^N(\tau _j -\bar{\tau })^2\right] ^{\frac{1}{2}}}. \end{aligned}$$

Note the correlation coefficient is always bounded by $[-\,1, 1]$, where $-\,1$ or 1, respectively, indicates a perfect negative or positive linear correlation.

### Error analysis

Numerical methods yield approximate results, where the numerical error *E* arises from the IMEX method and the Monte Carlo simulations. The IMEX time integration error $E_{\mathrm{ti}}$ is defined by

30 $$\begin{aligned} \Vert E_{\mathrm{ti}}\Vert = \Vert \hat{\tau }-\hat{\tau }^{\Delta t} \Vert \leqslant C\cdot \Delta t, \end{aligned}$$

here *C* represents a positive constant, $\hat{\tau }$ and $\hat{\tau }^{\Delta t}$ denote the real mean penetration time and numerical mean penetration time, respectively. The numerical result becomes accurate with the limitation of a sufficiently small time step $\Delta t$. Furthermore, the accuracy of the Monte Carlo simulations depends on the number of samples $N_\mathrm{s}$ and this error $E_{\mathrm{mc}}$ is achieved by

31 $$\begin{aligned} \Vert E_{\mathrm{mc}}\Vert = \Vert \hat{\tau }^{\Delta t}-\hat{\tau }_{N_\mathrm{s}}^{\Delta t}\Vert \simeq \frac{S_n}{\sqrt{N_\mathrm{s}}}, \end{aligned}$$

where $S_n$ denotes the sample standard deviation and $\hat{\tau }_{N_\mathrm{s}}^{\Delta t}$ is the sample mean as a result of $N_\mathrm{s}$ samples, which is $\hat{\tau }_{N_\mathrm{s}}^{\Delta t} = \frac{\sum _{j=1}^N\tau _j^{\Delta t}}{N_\mathrm{s}}$. Here $\tau _j^{\Delta t}$ denotes the penetration time of sample *j*, and the sample standard deviation is given by $S_n = \left[ \frac{\sum _{j=1}^{N_\mathrm{s}}(\tau _j^{\Delta t}-\hat{\tau }_{N_\mathrm{s}}^{\Delta t})^2}{N_\mathrm{s}-1}\right] ^{\frac{1}{2}}$. Note that this error decreases with increasing number of trials. Therefore, the total error *E* is given by

32 $$\begin{aligned} \begin{aligned} \Vert E\Vert = \Vert \hat{\tau }-\hat{\tau }_{N_\mathrm{s}}^{\Delta t} \Vert&\leqslant \Vert \hat{\tau }-\hat{\tau }^{\Delta t} \Vert + \Vert \hat{\tau }^{\Delta t}-\hat{\tau }_{N_\mathrm{s}}^{\Delta t}\Vert \\&\leqslant C\cdot \Delta t + \frac{S_n}{\sqrt{N_\mathrm{s}}}. \end{aligned} \end{aligned}$$

To keep the numerical approximation as accurate as possible, the time step should be small enough and number of samples should be sufficient large. Take the Monte Carlo simulations with six parameters as an example. If we fix all the six parameters and set the random walk parameter to zero, then the computation is fully deterministic. The time step is 0.0001 h and the constant C can be estimated using Richardson error estimation by

33 $$\begin{aligned} {\left\{ \begin{array}{ll} &{}\hat{\tau } = \hat{\tau }^{\Delta t} +C\cdot \Delta t \\ &{}\hat{\tau } = \hat{\tau }^{2\Delta t} +C\cdot 2\Delta t, \end{array}\right. } \end{aligned}$$

where $C = \frac{\hat{\tau }^{\Delta t}-\hat{\tau }^{2\Delta t}}{\Delta t} = 133$, which is a mean value of ten times calculations. With 10,000 Monte Carlo (with sampling in the six parameters and using random walk) samples, the analytical error analysis can be derived as,

34 $$\begin{aligned} \begin{aligned} \Vert E\Vert&\leqslant 1.33\times 10^{-2} + \frac{0.0687}{\sqrt{10{,}000}} \\&\simeq 1.40\times 10^{-2}, \end{aligned} \end{aligned}$$

where 0.0687 is the sample standard deviation $S_n$. Therefore, the total error in the Monte Carlo simulations with six parameters is bounded by 0.014 h.

**Table 2:** Parameter values

Constant	Notation	Value	Unit	Source
Radius of a circular cell in 2D	*R*	12.5	$\upmu \mathrm{m}$	(Champion and Mitragotri 2006)
Radius of a spherical cell in 3D	*R*	10	$\upmu \mathrm{m}$	(Champion and Mitragotri 2006)
Radius of a circular nucleus in 2D	$R^{ n}$	5	$\upmu \mathrm{m}$	(Friedl et al. 2011)
Radius of a spherical nucleus in 3D	$R^{ n}$	8	$\upmu \mathrm{m}$	(Friedl et al. 2011)
Cell deformation relaxation	$\alpha$	250	$\mathrm{h}^{-1}$	estimated
Nucleus deformation relaxation	$\alpha ^n$	2500	$\mathrm{h}^{-1}$	estimated
Diffusivity of the chemokine	*D*	3600	$\upmu \mathrm{m}^{2}/\mathrm{h}$	(Jayaraman et al. 2001)
Mobility of points on cell membrane	$\beta$	60	$\mathrm{h}^{-1}$	(Vermolen and Gefen 2012)
Secretion rate of the chemokine	$\gamma _\mathrm{s}$	1.2 $\times 10^6$	$\mathrm{mol/h}\, \upmu \mathrm{m}^3$	(Savinell et al. 1989)
Time step in 2D	$\Delta$t	0.0001	h	(Pinner and Sahai 2008)
Time step in 3D	$\Delta$t	0.01	h	–
Number of nodes on a 2D cell	*N*	30	–	–
Number of circles on a 3D cell	$N_\mathrm{c}$	30	–	–

## The numerical simulations

First, we describe the simulations in which one cell migrates toward the gradient of an increasing stimulus along obstacles in 2D and 3D. Subsequently, this deformation model of cell and its nucleus is applied to a simplified cancer metastasis phenomenon. Furthermore, six parameters are studied and analyzed by Monte Carlo simulations.

### Parameter values

Most often the experimental parameter values are not available to us, therefore estimating input values based on experimental literature is essential. For example, we use 10 $\upmu \mathrm{m}$ in 2D and 16 $\upmu \mathrm{m}$ in 3D for diameters of the nucleus referring to the work by Friedl et al. (2011), where the diameter of the nucleus varies from 10–20 $\upmu \mathrm{m}$ in 2D and 5–15 $\upmu \mathrm{m}$ in 3D. Analogously, other default input values are listed in Table 2, as well as the sources from the literature whenever possible.

### Cell migration along a rigid object in 2D and 3D

#### One cell migrating along a rigid object in 2D

In solid tumors, cell migration shows trends in its direction according to the presence of chemotactic gradients or other external cues. Since there are many parallels existing in the mechanisms underlying the movement of cancer cells and immune cells within tissues as well as in the blood circulation (Pinner and Sahai 2008), the modeled cell can be an immune cell with a chemical source of antigen or a cancer cell with a source of oxygen or substrate/ECM stiffness.

**Fig. 3:**
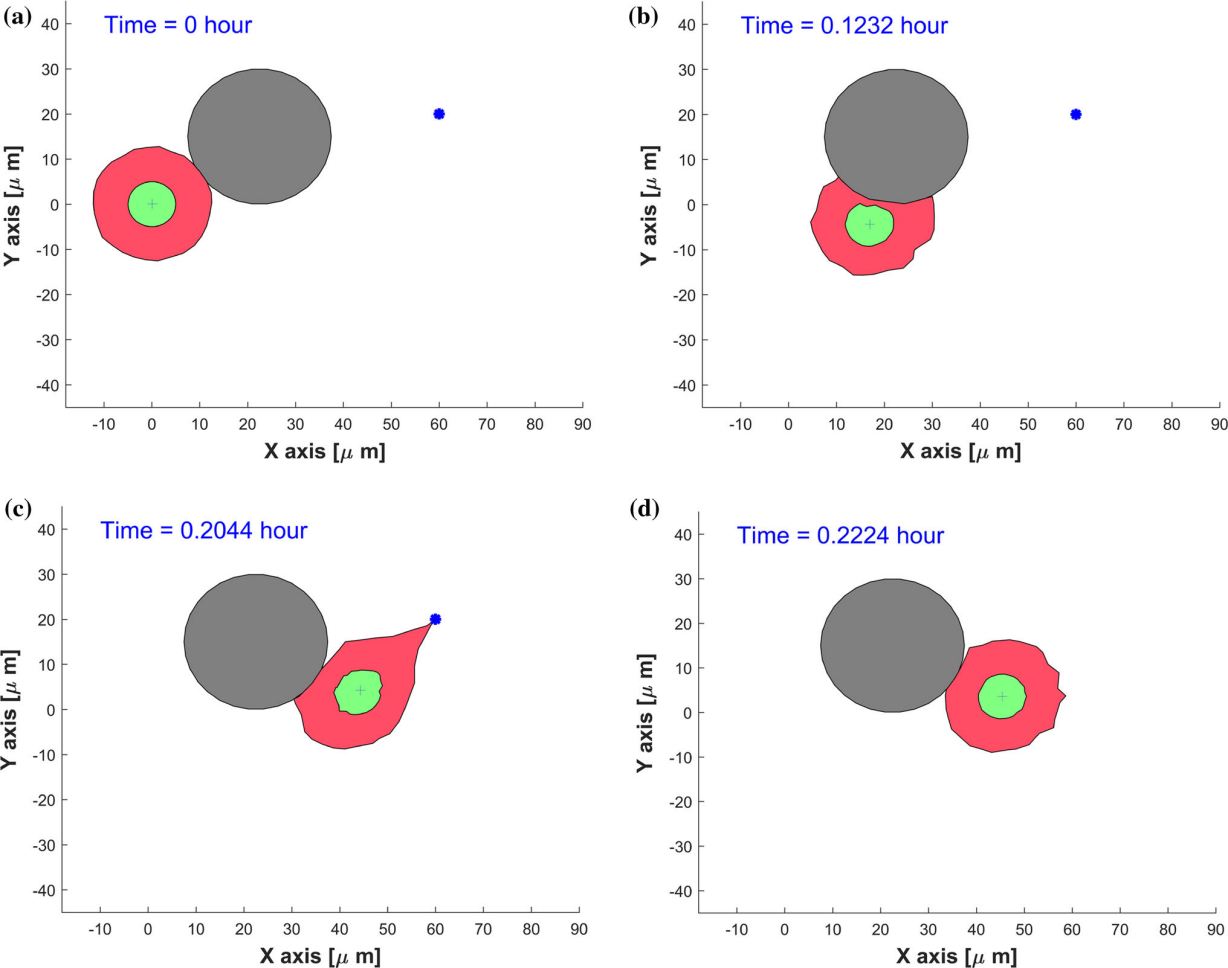
Consecutive snapshots of one cell migrating along a rigid obstacle in a 2D simulation. The cell, nucleus and obstacle are visualized by red, green and gray colors, respectively. A blue asterisk denotes a source secreting a chemokine with the secretion rate of $2\times 10^5\,\mathrm{mol/h}\,\upmu \mathrm{m}^3$. The CPU time of this model takes 2.20 s

The cell moves according to the gradient of chemokine. Snapshots at different stages of the migration are shown in Fig. 3, where the red, green and gray objects visualize the cell, nucleus and a rigid obstacle, respectively. Furthermore, the signal source location is represented by an asterisk. To pass a stiff barrier or overcome an obstacle, the migrating cell has to reshape and adapt the mechanostructure of the cytoplasm and the membrane. That is done via exerting contractile forces or withstanding the stresses from neighbor cells, which are mediated by the cell cytoskeleton (Brunner et al. 2006). According to the experimental observation of Brunner et al. (2006), one migrating cell could push a small obstacle upward by exerting forces and crawl underneath this obstacle. Given a larger obstacle in our simulation, the cell and nucleus are more likely to crawl along the rigid boundary by morphological adjustments to different extents. Ultimately, the cell and nucleus are able to return to their initial shapes due to cell polarity once the source is no longer active.

#### One cell moving along a rigid object in 3D

**Fig. 4:**
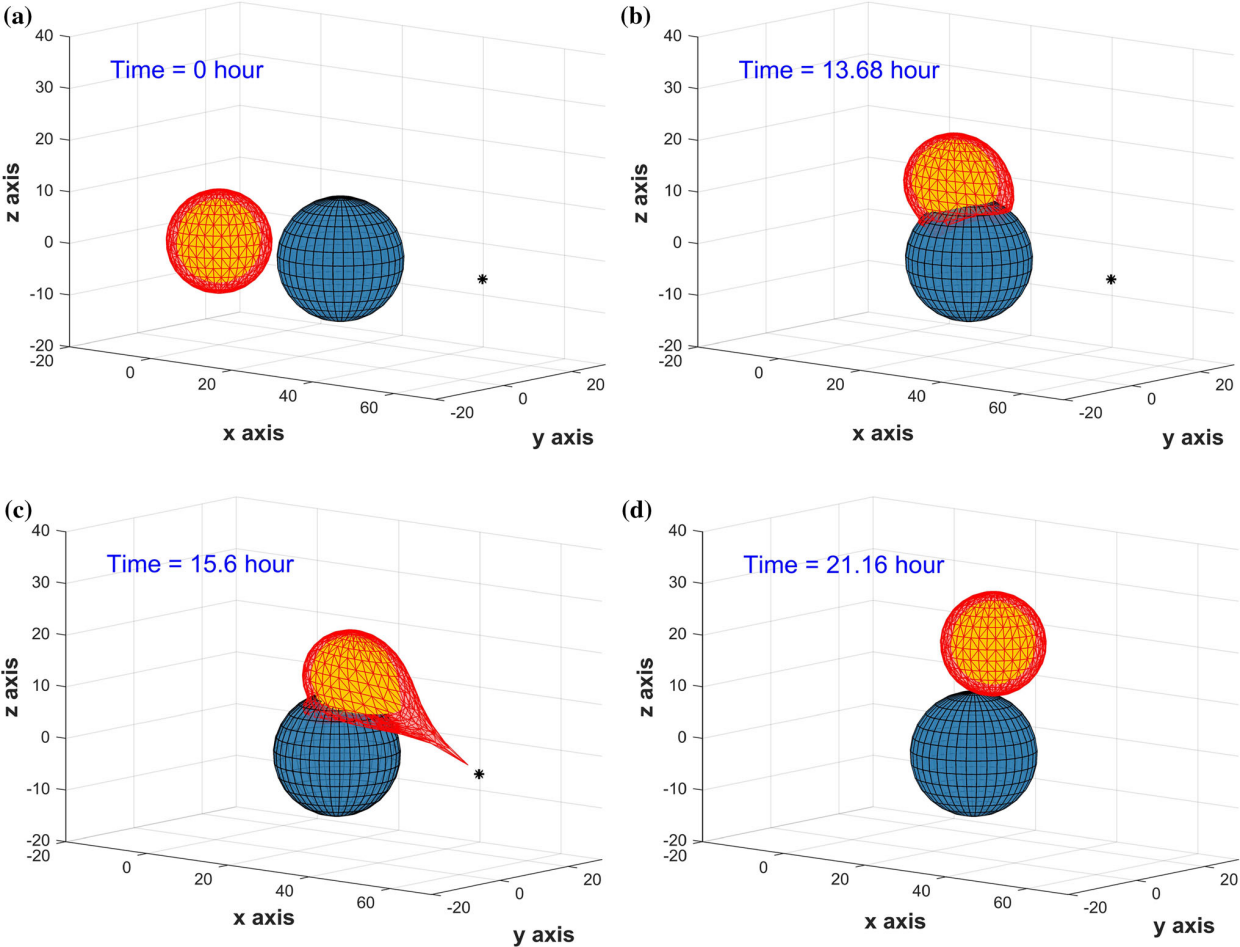
Consecutive snapshots of one cell migration along a rigid obstacle in 3D simulation. The cell, nucleus and obstacle are visualized by red, yellow and blue colors, respectively. A black asterisk denotes any type of sources. The CPU time of this model is 21.77 s

In three-dimensional interstitial tissues, cells typically utilize one of two mechanisms for invasion: mesenchymal or amoeboid, respectively, involving degradation of the surrounding ECM or squeezing through sub-cell-size pores in the ECM; these mechanisms require, respectively, proteinases that can degrade the ECM or deformations of the cell shape. (Friedl et al. 2011). To simplify the problem and make it time-efficient to solve, we only consider the mechanical deformability of the cell in this model rather than deformability of both the environment and the cell. The degradation of the ECM is hence modeled implicitly in the $\beta$-term in Eq. 3. Note that if the ECM decay-rate would be zero, then the $\beta$-parameter would be zero as well. Hence the $\beta$-parameter accounts for the decay of ECM and the mobility of the node. In a future study, the decay process of the ECM could be modeled more explicitly so that the migration and deformation process of the cell can be modeled to be rate-determined by the slowest process. We model a 3D cell with a spherical equilibrium geometry that travels over an obstacle toward a source that secretes a chemokine, e.g., for immune cells the source may be a pathogen. In Fig. 4, consecutive snapshots of a 3D cell that reaches a source and engulfs are shown. It can be seen that the cell deforms mechanostructurally and that the cell shape returns to its equilibrium spherical shape once the stimulus has been removed. Note that the cell is still attached to the obstacle once the source has disappeared. Due to this mechanical attachment and cell elasticity, the cell deforms back to its equilibrium and thereby pushes itself away from the obstacle such that there is only attachment at one point of the cell boundary to the obstacle. This is a characteristic of the current model in which steady-state adherence has been neglected. The figures illustrate how the model takes into account the hard mechanical impingement between the cell and the rigid obstacle.

In general, dimensionality does not affect the expected numerical result in this case. Furthermore, the computational time of a 2D model is much shorter as a result of the need for fewer gridpoints on the boundaries of the cell and nucleus, and thereby we use 2D model for further application and analysis in this work.

### Application to cancer metastasis in 2D

There are preexisting openings (pores, fiber-like or channel-like tracks) in ECM that enable cancer cells to migrate with an independence of MMP’s (Paul et al. 2017). In this section, we apply the model to the transmigration of cancer cells through pores and channels to migrate from one part to another part of the tissue without degrading ECM.

#### Simulation on penetration of a cell through a cavity

**Fig. 5:**
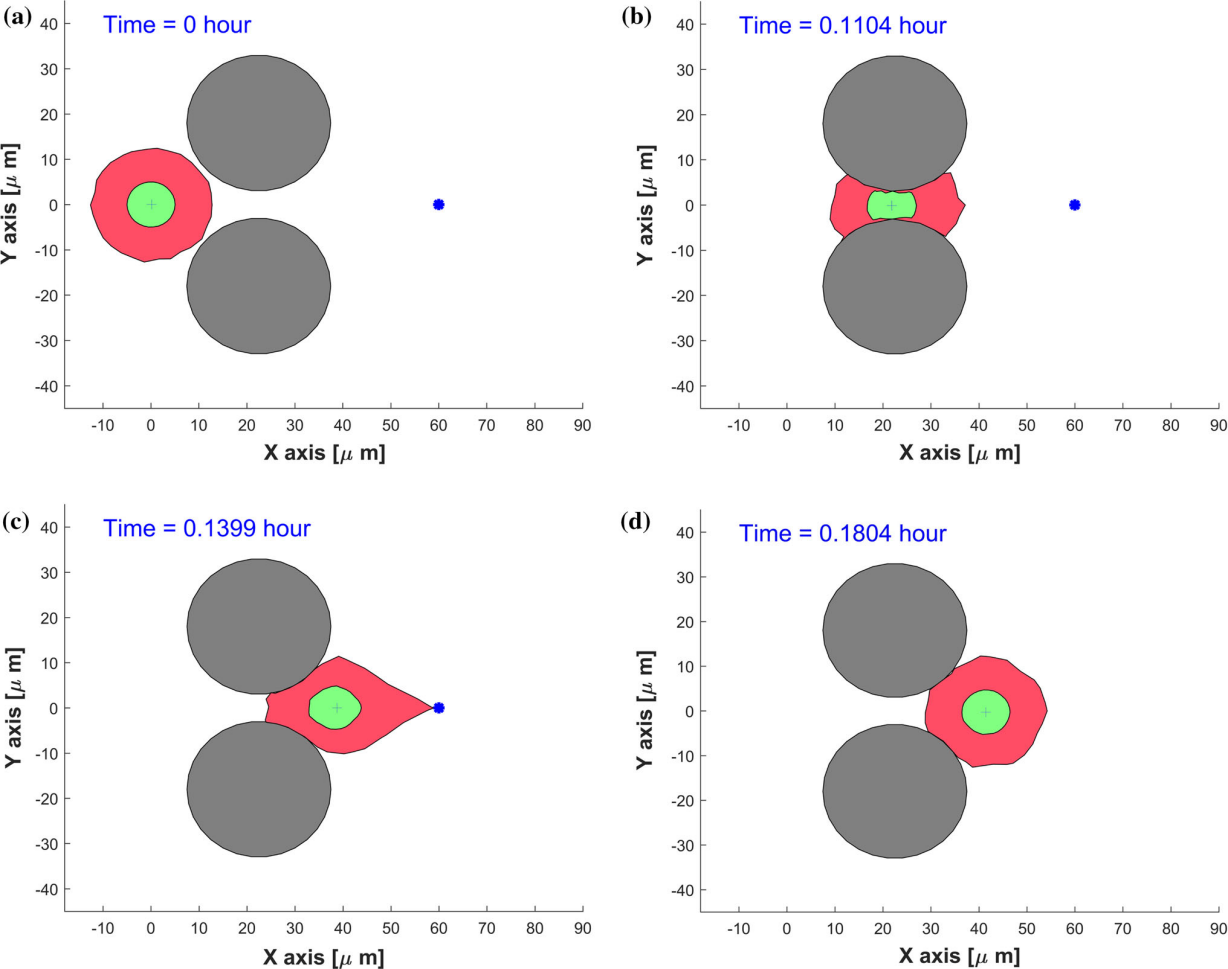
Consecutive snapshots of one cell penetration a cavity made of two obstacles in 2D simulation. The cell, nucleus and obstacles are visualized by red, green and gray colors, respectively. A blue asterisk denotes any type of sources. The CPU time of this model is 2.18 s

We initially consider a single cell penetrating through a cavity, which is formed by two circular obstacles, without secreting proteolytic enzymes and remodeling the ECM; i.e., the cell migration is assumed to utilize the amoeboid mode. The initial state is shown in Fig. 5 (top-left). The cell is attracted to an imaginary source (indicated by the blue asterisk) that releases a chemokine or a ECM stiffness signal. The migration of the cell is directed up the gradient of the chemokine, and it is limited by the presence of the two physical obstacles. Further, it can be seen that the cell is mechanically compressed as a result of its shrinkage due to its migration through the cavity and the nucleus deforms whenever the size of the pore is smaller than the size of the nucleus, see Fig. 5. As soon as the cell exits the constriction and is no longer mechanically compressed, the nucleus returns to its equilibrium circular shape. Once the source has been engulfed, the cell shape returns to its equilibrium circular shape. The model only incorporates temporary adherence to the obstacle, no permanent adherence. After disappearance of the source, only restoration of the cell shape is modeled.

#### Simulation on penetration of a cell through a tube channel

**Fig. 6:**
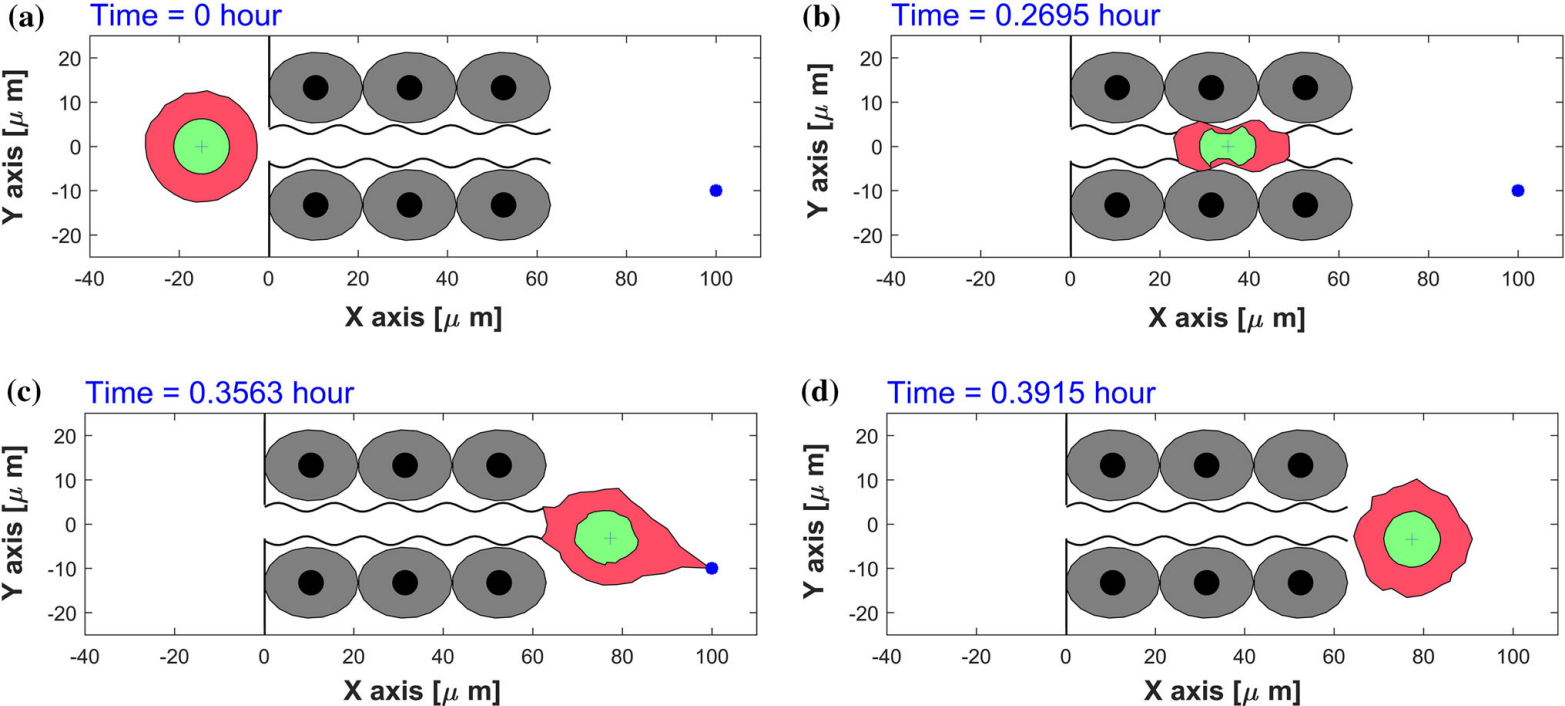
Consecutive snapshots of one cell penetration through an endothelial cell wall in 2D simulation. The migrating cell, nucleus and endothelial cells are visualized by red, green and gray colors, respectively. A blue asterisk denotes any type of sources. The CPU time of this model is 6.05 s

Cell deformation is normally studied in vitro by using microfluidic devices(Mak et al. 2013; Paul et al. 2016; Myrand-Lapierre et al. 2015). In the latter work, discord-shaped red blood cells have been shown to be able to repeatedly deform when penetrating through microcapillaries with a diameter of 2.5 $\upmu \mathrm{m}$ or even less. As mentioned in Sect. 2.3, there are abundant preexisting fiber-like and channel-like tracks formed by the alignments of the collagen architecture in interstitial tissues and organs, which guide or inhibit cell migration (Wolf et al. 2009; Paul et al. 2017). In Fig. 6, a schematic representation of an endothelial cell wall with a channel of approximate 10 $\upmu \mathrm{m}$ in width is depicted (Paul et al. 2017). This value is considered here to guarantee that the cell is able to penetrate through it in most cases.

Mechanical boundaries could regulate some biomedical processes and Mak et al. (2013) demonstrate that if the confined dimensional modulation of a microfluidic device has a mechanical barrier smaller than the cell nucleus, then metastatic breast adenocarcinoma cells likely deform in elongated morphological states and invade distinct sites. Here, taking mechanical boundaries into account, we use the trigonometric function (from equation (15)) to simulate the different roughnesses through changing the value of parameter $\epsilon$ and $\omega$. A highly rough boundary of the channel is defined if the perturbation [see Eq. (15)] has a high frequency or/and a big amplitude, which is determined by the surface of the endothelial cells. Whereas a lower frequency (also a lower amplitude) as we depict in Fig. 6 could show where each cell is located. The discrepancy between the endothelial cellular surfaces and the channel through which the cancer (or immune) cell migrates, could be a consequence of the extracellular matrix around the cells. Then the boundary of the channel can have various roughnesses, which combined with other parameters, are analyzed by using Monte Carlo simulations based on this model. Moreover, this model is incorporated with Poisseuille flow to simulate the micro blood flow referring our work (Chen et al. 2018). To investigate how the cell speed changes in the current scenario, the speed evolution with the respect of time is plotted in Fig. 8a without the perturbation of vector Wiener process. As we expected, the cell speed slows down when it starts to squeeze the opening and subsequently accelerates to move toward the emitting source. When the $\tau$ equals approximate 0.37 hour, the instantaneous speed reaches a peak and drops to zero after the engulfment of the source and cell shape recovery. During the transmigration in the tube, the cell migrates with a speed vibrating up and down at 200 $\upmu \mathrm{m/h}$, which is in the range 1–5 $\upmu \mathrm{m/min}$ for the typical speed of amoeboid movement observed in vivo in the work (Pinner and Sahai 2008). Moreover, the cell speed can be controlled under various conditions, like the number of emitting sources, the diffusion coefficient, cell mobility.

#### Simulation on cancer metastasis

**Fig. 7:**
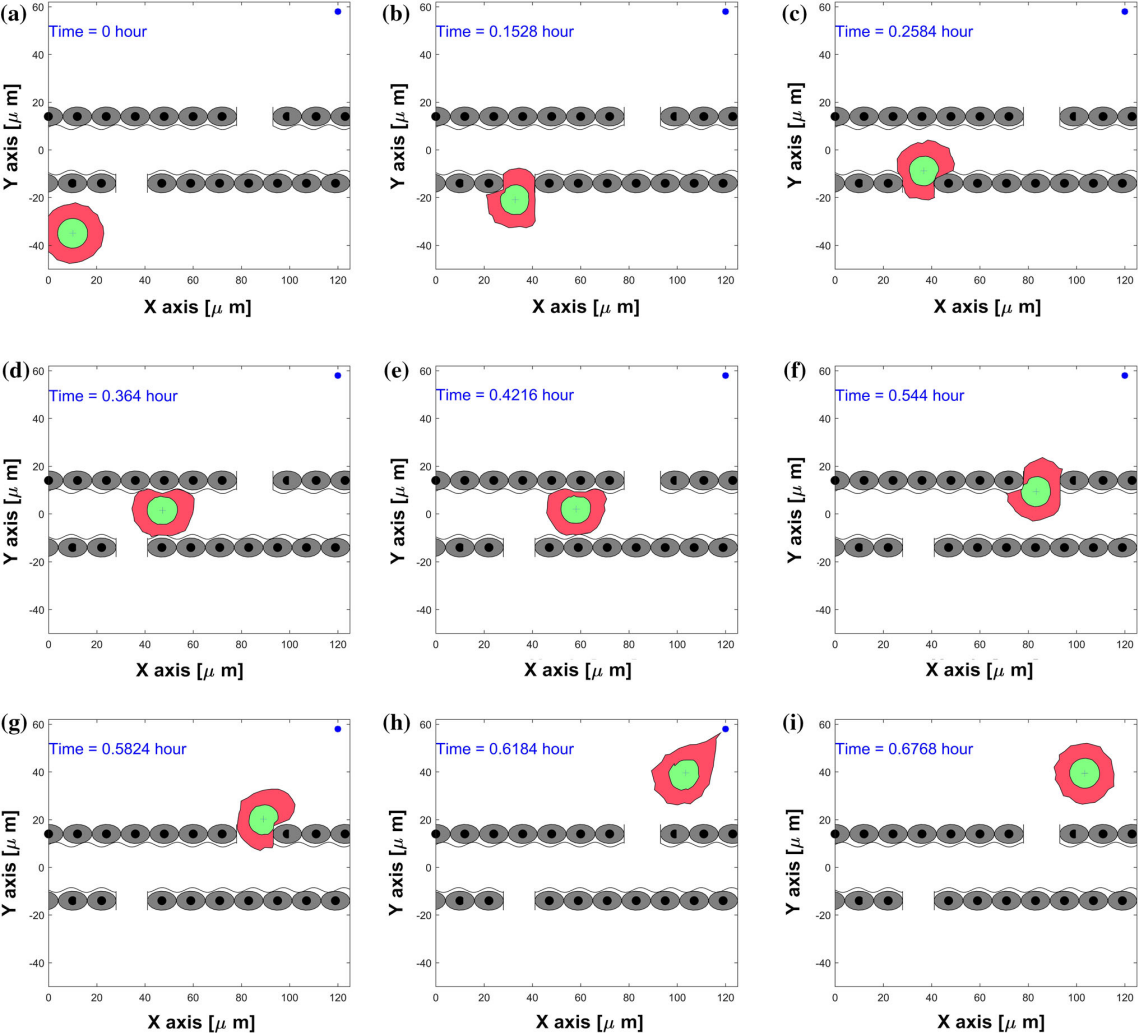
Consecutive snapshots of one cell about intravasation and extravasation of a blood or lymphatic vessel in 2D simulation. The migrating cell, nucleus and the vessel are visualized by red, green and gray colors, respectively. A blue asterisk denotes any type of sources. The CPU time of this model is 7.30 s

**Fig. 8:**
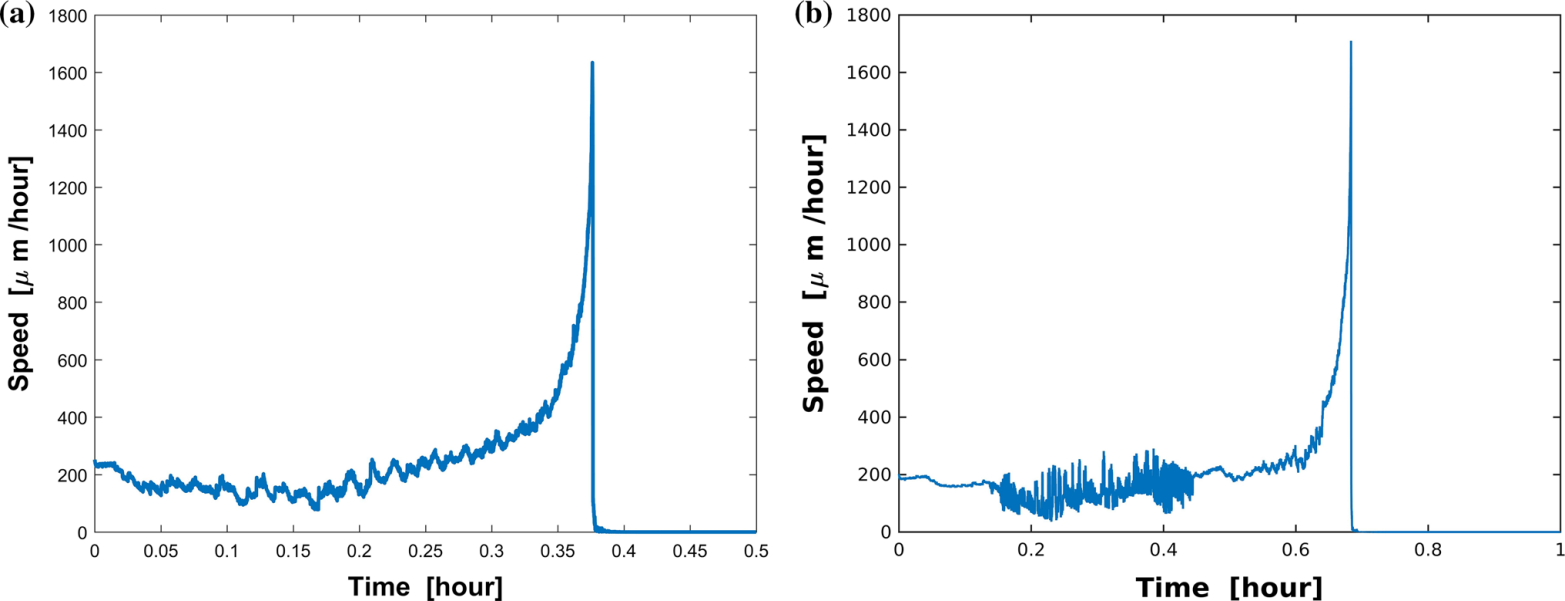
**a** The cell speed evolution in cell penetration model (Fig. 6); **b** the cell speed evolution in cell metastasis model (Fig. 7)

Immune cells and cancer cells similarly deform in chemically or mechanically induced locomotion. The work by Springer (1994) reports that leukocytes can attach to the wall of a blood vessel by binding to adhesion molecules of the endothelial cells, subsequently the leukocytes flatten themselves, and then squeeze through openings which are much smaller than themselves among the endothelial cells. Analogously, metastatic cells utilize similar mechanisms when intravasting into or extravasating out of blood vessels. Cancer metastasis is a multi-step cascade that can be divided into the following steps, (1) escape from the primary tumor site; (2) survive transit in the bloodstream or lymphatic vessels after successful intravasation; (3) disseminate and extravasate subsequently; (4) start to proliferate and colonize secondary sites at distant organs (Chambers et al. 2002a; Kopfstein and Christofori 2006). We attempt to simulate the steps of intravasation and extravasation and several consecutive snapshots showing the shape changes of cell and nucleus are provided in Fig. 7, where a schematic diagram of a capillary-sized channel is depicted. In order to get around hypoxia (or lack of nutrition) as a result of competitive growth in cancer cell colonies or as a response to a stiffness gradient, metastatic cells show migratory exploratory behavior toward regions outside the cology they reside in. This migration can be inspired by gel-stiffness-dependent differences in traction forces or strain energies in Massalha and Weihs (2017). Therefore cancer cells are capable of penetrating through small openings in endothelium. This process is highly inefficient, and during this dissemination, the majority of cancer cells would die and only $<\,0.02\%$ of them are able to seed at distant sites successfully (Celià-Terrassa and Kang 2016; Luzzi et al. 1998). Analogously, the cell speed evolution of this model is shown in Fig. 8b, and the speed is around 200 $\upmu \mathrm{m/h}$ in the channel and reaches a peak instantaneously when the cell gets close to the source. The reason for this peak is the singularity in Eq. (2) at the position of the source, which gives a very large gradient of the concentration near the source. This peak could be regularised by either adding a time-dependency (through an analytic solution or through a numerical solution of the concentration) or by replacing the chemotaxis by a factor such that the velocity stays bounded. All these approaches make the model more complication and since the objective was a construct a simple model, this has been omitted. At the final stages, the speed decreases to zero due to lack of attraction signals and the vector Wiener process. We also remark that the large variations in Fig. 8b are caused by the cell having to pass through the apertures and having to migrate along the wall of the channel. This interaction between the cell boundary and obstacle causes the switch between repulsion and migration along the tangent of the obstacle and attraction as a result of a component normal to the tangent of the boundary of the obstacle. This effect of this discontinuous switch mechanism can only be inhibited by choosing a smaller time step.

### Parameter study with Monte Carlo simulations

If certain input values contain uncertainties, Monte Carlo simulations could be a way to evaluate the impacts of output. This method enables us to estimate of the impact from variables ranging from various statistical distributions like Pareto, uniform, normal, lognormal, Chi-square, exponential (Mooney 1997). Furthermore, Monte Carlo simulations have been used over a spectrum of systems, which is typically concluded in following four steps, (1) generate the input random values based on their probability distribution functions; (2) calculate samples; (3) repeat the above-mentioned steps with a number of trials $N_\mathrm{s}$; (4) calculate the mean and construct a relative frequency distribution of the simulated results (Mooney 1997; Mahadevan 1997). Furthermore, one can estimate the correlation between the various input and output parameters.

The model introduced in Sect. 4.3.2 is used in Monte Carlo simulations, with the channel boundary of 60 $\upmu \mathrm{m}$ in length and approximately 10 $\upmu \mathrm{m}$ in width. The transit time interval that starts once one of the cell’s boundary points enters the channel and lasts until the last point exits the channel is defined as the penetration time $\tau$. In this section, the influences of several parameters on the penetration time $\tau$ are investigated.

**Fig. 9:**
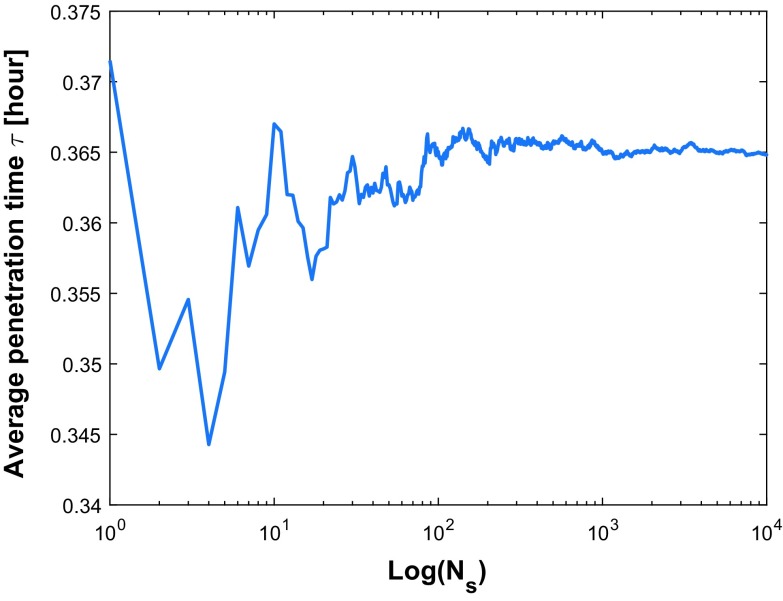
Sample quantity test for convergence of average penetration time $\tau$. The penetration time is in hours

As we discussed in Sect. 3.4, the accuracy of the simulation result depends on the number of samples. To achieve an accurate approximation, the number of samples is tested that is shown in Fig. 9.

Note that the axes represent the logarithm of sample count and the mean of transit time, respectively. If the sample count in the Monte Carlo simulations is too small, then the average penetration time has not yet converged (see Fig. 9 for $N_\mathrm{s} < 200$). We observe that using 10,000 samples only gives very small fluctuations of the average penetration time (see Fig. 9). The result has converged sufficiently to approximate 0.356 h. However, to evaluate the uncertainty of input data quantitatively, 10,000 samples are chosen in our simulation which give acceptable computation times in the order of hour. Using equation (31), the Monte Carlo error is estimated by $\Vert E_{\mathrm{mc}}\Vert = \Vert \hat{\tau }^{\Delta t}-\hat{\tau }_{N_\mathrm{s}}^{\Delta t}\Vert \simeq \frac{S_n}{\sqrt{N_\mathrm{s}}}$.

#### Monte Carlo simulations on parameters *D*, $\beta$, $\alpha$, $\alpha ^n$

We start with the Monte Carlo simulations on four input parameters which are the diffusion coefficient of the chemokine *D*, cell point mobility $\beta$, cell deformation relaxation $\alpha$ and the nucleus deformation relaxation $\alpha ^n$. We sample them from the normal distribution, then they can be generated by Eq. (28) with the default values in Table 3.

**Fig. 10:**
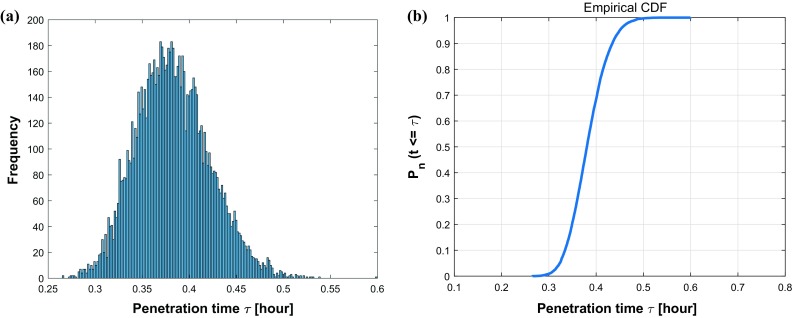
The histogram (**a**) and CDF plot (**b**) of cell penetration time $\tau$ in Monte Carlo simulations on parameters *D*, $\beta$, $\alpha$, $\alpha ^n$

The mean value of each is the same as the value in Table 1 and corresponding standard deviation reflects the degree of dispersion among samples. The values have been chosen mathematically based on an extensive testing. In Fig. 10, we plot a histogram of 10,000 samples as well as a cumulative distribution function (CDF) of the estimated probability of penetration time $\tau$. Thence, the x-axis denotes the consecutive variable penetration time $\tau$ and the y-axis represents the frequency of occurrence or the probability $P_n (t \le \tau )$ of the corresponding variable depending on the chart considered in Fig. 10.

**Table 3:** Parameter values

	*D*	$\beta$	$\alpha$	$\alpha ^n$
Value	$N \sim (3600, 30^2)$	$N \sim (60, 3^2)$	$N \sim (250, 40^2)$	$N \sim (2500, 125^2)$

**Fig. 11:**
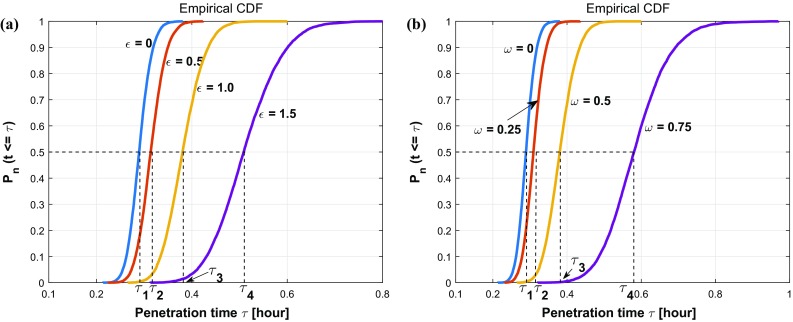
**a** Compares the CDF plots of cell penetration time $\tau$ in terms of various $\epsilon$ ($\epsilon = 0, 0.5, 1.0, 1.5 \,\upmu \mathrm{m}$) with a fixed $\omega$ value ($\omega = 0.5\, \upmu \mathrm{m}^{-1}$). **b** Compares the CDF plots of cell penetration time $\tau$ in terms of various $\omega$ ($\omega = 0, 0.25, 0.5, 0.75 \,\upmu \mathrm{m}^{-1}$) with a fixed $\epsilon$ value ($\epsilon = 1.0 \, \upmu \mathrm{m}$)

**Fig. 12:**
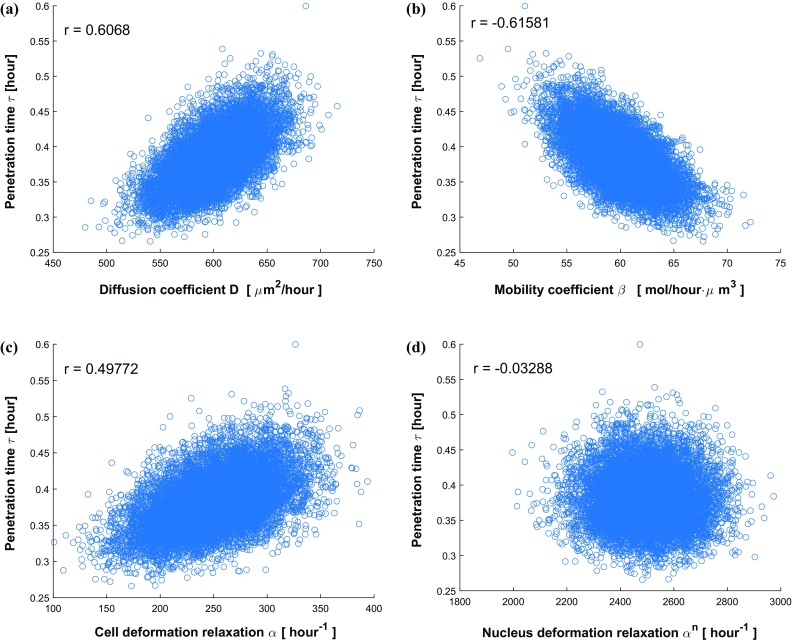
Scatter plots about cell penetration time $\tau$ with respect to various variables *D*, $\beta$, $\alpha$, $\alpha ^n$

Taking different roughnesses of the channel boundary into consideration, various values of $\epsilon$ ($\epsilon = 0, 0.5, 1.0, 1.5\,\upmu \mathrm{m}$) and $\omega$ ($\omega = 0, 0.25, 0.5, 0.75\, \upmu \mathrm{m}^{-1}$) are set and compared in Fig.11. The $\epsilon$ parameter manifests the magnitude of vertical fluctuation, i.e., the amplitude; while $\omega$ determines the frequency of the fluctuations of boundary. A smooth boundary has a small $\omega$ value, then one cell is able to move through it much faster than through a rough channel. In Fig.11a, we observe four cumulative distribution functions with different slopes $f(\tau )$, which represents the probability density. Thus, any probability $P_n$ of a time interval $[\tau -\frac{\Delta t}{2}, \tau +\frac{\Delta t}{2}]$ occurring can be calculated by the formula $P_n(\tau -\frac{\Delta t}{2}\le \hat{\tau } \le \tau +\frac{\Delta t}{2}) \approx f(\tau ) \cdot \Delta t$. Conversely, with the same probability, taking $P_n = 0.5$ for an example, we can get the information about the transit time of one cell with 50% probability in various conditions, where $\tau _1(\epsilon = 0 \, \upmu \mathrm{m})< \tau _2(\epsilon = 0.5 \, \upmu \mathrm{m})< \tau _3(\epsilon = 1.0 \, \upmu \mathrm{m}) < \tau _4(\epsilon = 1.5 \, \upmu \mathrm{m})$. Analogously, Fig.11b showing four cumulative distribution functions of the penetration time $\tau$ are compared under different conditions with varying $\omega$. With 50 % probability, one cell takes penetration time $\tau _1$ with a straight boundary $\omega = 0 \, \upmu \mathrm{m}^{-1}$, the penetration time rises to $\tau _2$, $\tau _3$ and $\tau _4$ with the increase in roughnesses $\omega = 0.25 \, \upmu \mathrm{m}^{-1}$, $\omega = 0.5 \, \upmu \mathrm{m}^{-1}$ and $\omega = 0.75 \, \upmu \mathrm{m}^{-1}$, respectively. In conclusion, both the standard deviation in the arrival times and the mean arrival time increase with increasing values of $\epsilon$ and $\omega$. Subsequently, we fix the roughness parameter values to $\epsilon = 1.0 \, \upmu \mathrm{m}$ and $\omega = 0.5 \, \upmu \mathrm{m}^{-1}$, then the impacts of four parameters $D, \beta , \alpha , \alpha ^n$ on the penetration time $\tau$ are investigated and the correlation analysis are shown in Fig. 12. Based on the results, there is some positive correlation between the penetration time $\tau$ and both *D* and $\alpha$ with correlation coefficient *r* equal to 0.6068 and 0.49772, respectively. Moreover, $\beta$ has a negative linear correlation with $\tau$, whereas, the nucleus deformation relaxation has no obvious correlation with penetration time in this situation. However, the nucleus is the stiffest cellular component, which inhibits the confined cell migration if the pore diameter in the ECM is below a critical threshold (Wolf et al. 2013; Davidson et al. 2014). Therefore, the correlation between the penetration time and nucleus stiffness is expected to be highly positive if the width of channel is smaller than a critical threshold.

#### Monte Carlo simulations on parameters $\epsilon$ and $\omega$

We next analyze the other two parameters $\epsilon$ and $\omega$, which reflect the amplitude and frequency of the channel boundary. Suppose $\epsilon$ and $\omega$ are too large, i.e., $\epsilon ,\omega >2$ in our simulations, the trigonometric functions probably would trap migrating cells due to sharp peaks or corners. Therefore, $\epsilon$ and $\omega$ are generated carefully with uniform normal distribution by the following equation,

35 $$\begin{aligned} {\left\{ \begin{array}{ll} \epsilon &{}\sim \quad U (0.5, \ 1.5),\\ \omega &{}\sim \quad U (0, \ 0.6). \end{array}\right. } \end{aligned}$$

**Fig. 13:**
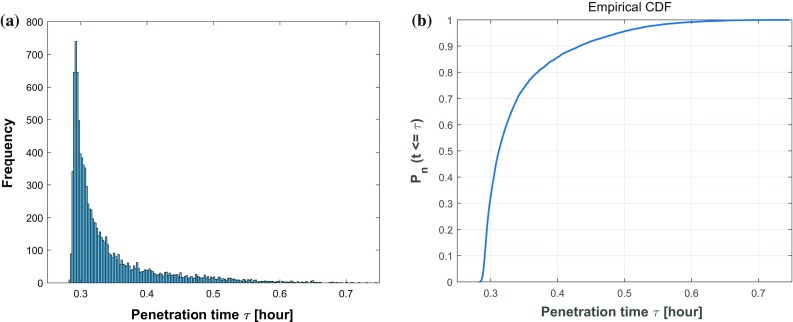
The histogram (**a**) and CDF plot (**b**) of cell penetration time $\tau$ in Monte Carlo simulations on parameters $\epsilon$ and $\omega$

This above equation guarantees the value of $\epsilon$ and $\omega$ are uniformly distributed and bounded by $(0.5, \ 1.5)$ and $(0, \ 0.6)$. Based on the 10,000 samples, Fig. 13a shows the corresponding histogram, which looks like a lognormal chart and fits from a qualitative point of view with the experimental results by Abuhattum and Weihs (2016), where the migration speeds of single preadipocytes without chemoattractants follow a lognormal distribution. A cumulative percentage of the number of occurrences regarding the cell penetration time $\tau$ is plotted in Fig. 13b.

**Fig. 14:**
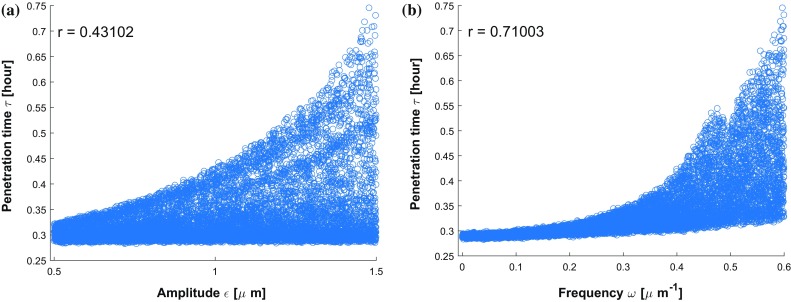
Scatter plots about cell penetration time $\tau$ with respect to various variables $\epsilon$ and $\omega$

Analogously, scatter diagrams about $\epsilon$ and $\omega$ with penetration time $\tau$ indicating their correlations are shown in Fig. 14. With the increase in roughness, one cell travels a longer time to penetrate the channel in most cases. Furthermore, the increment of $\omega$ makes a contribution to the total travel time of one cell. This is also reflected by the correlations of $r = 0.4310$ and $r = 0.7100$ between the penetration time and $\epsilon$ and $\omega$, respectively.

#### Monte Carlo simulations on parameters *D*, $\beta$, $\alpha$, $\alpha ^n$, $\epsilon$ and $\omega$

**Fig. 15:**
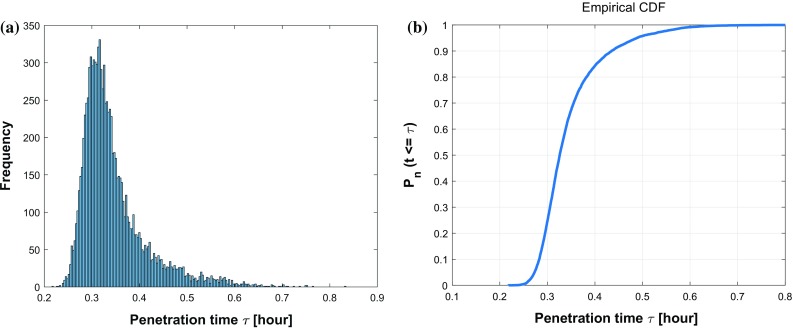
The histogram (**a**) and CDF plot (**b**) of cell penetration time $\tau$ in Monte Carlo simulations on parameters *D*, $\beta$, $\alpha$, $\alpha ^n$, $\epsilon$ and $\omega$

To test the essential variables simultaneously, all six parameters *D*, $\beta$, $\alpha$, $\alpha ^n$, $\epsilon$ and $\omega$ are analyzed by Monte Carlo simulations. The histogram of the penetration time $\tau$ is shown in Fig. 15a which can be fitted to a lognormal distribution. Furthermore, a CDF result is shown based on a sample of 10,000 times simulations in Fig.15b.

**Fig. 16:**
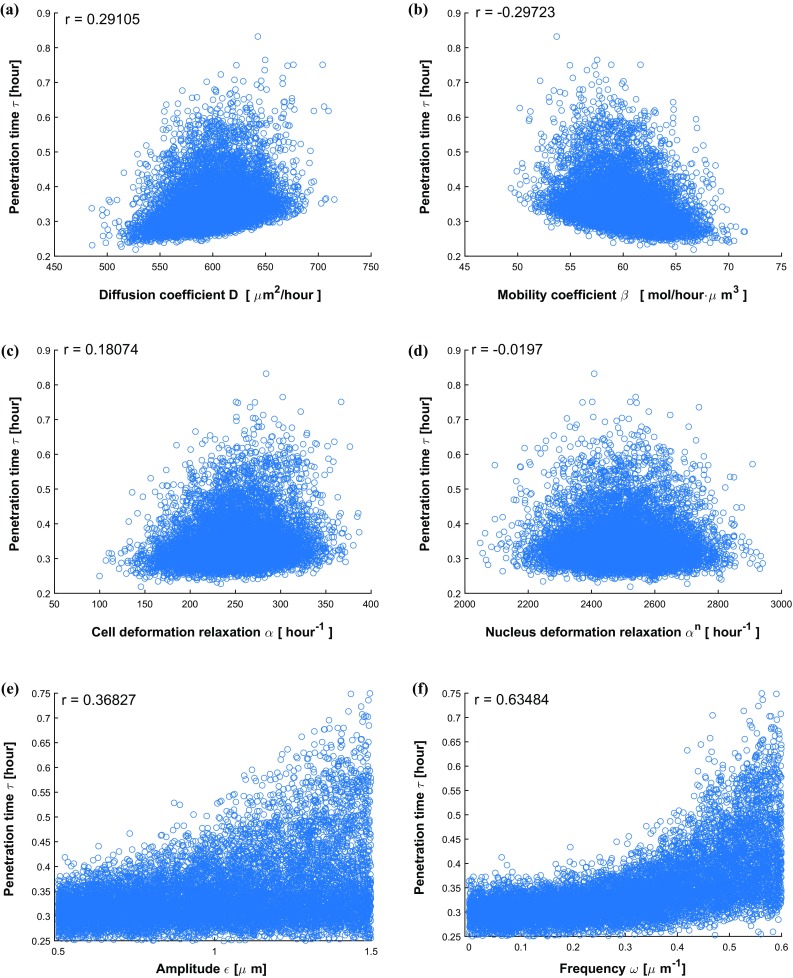
Scatter plots about cell penetration time $\tau$ with respect to various variables D, $\beta , \alpha ,\alpha ^n, \epsilon \,\, \mathrm{and}\,\, \omega$

To investigate the impacts of variables on output results and analyze the correlations of each variable with penetration time $\tau$, a couple of scatter plots are shown in Fig. 16, respectively. Adding some control variables that are statistically distributed yields more uncertainty to the system. The increase in uncertainty generally decreases the correlation. Therefore, in current simulation of six parameters, the correlation of parameters *D*, $\beta$, $\alpha$, $\epsilon$ and $\omega$ with time $\tau$ decrease slightly compared with the simulations with the variation of four parameters. The correlation between $\tau$ and $\alpha ^n$ is still negligible. Further, Fig. 16 shows that the roughness ($\epsilon$ and $\omega$) dominantly influences the cell travel time.

## Discussion and conclusions

In this work, we develop a cell-based model to describe the morphological evolution of the cell and nucleus in a phenomenological way. The cell cytoskeleton spanning between the nucleus and the cell membrane is simulated by 30 springs. As we expected, an immune cell or a single cancer cell can deform according to the specific obstacles or paths when it encounters a stiff obstacle in a 2D or 3D environment. Compared with some existing models, e.g., a model investigating the role of nucleus deformation in the cell deformation under different geometrical and fluid flow conditions (Serrano-Alcalde et al. 2017) and a three-dimensional model describing nucleus mechanics during cell migration and deformation (Giverso et al. 2018), one of the major advantages of our modeling is its efficiency regarding CPU time, which enables to carry out Monte Carlo simulations for evaluation of parameter sensitivity. A further merit of the current model is its simplicity. If one is able measure the velocity of points on the surface of the cell under the influence of (the gradient of) a generic (being a concentration or a stiffness for instance) signal, then the $\beta$-parameter can be determined. If one further is able to measure the retraction speed on the boundaries of the cell and the nucleus once the signal has disappeared, then it can fit the $\alpha$ parameters.

The uncertainties in the input values necessitate us to study the impact of uncertainty by carrying out Monte Carlo simulations. With 10,000 samples, the correlations of each variable *D*, $\beta$, $\alpha$, $\alpha ^n$, $\epsilon$ and $\omega$ with cell penetration time $\tau$ are analyzed. The results show that $\alpha ^n$ has no significant correlation with the penetration time in current situation, where the reason probably is the low range of parametric values in our simulations. A larger range, with variations over a lognormal distribution could give a higher correlation. The use of very high values of $\alpha ^n$ in the model when the cell is penetrating through an aperture needs more investigation. Moreover, Serrano-Alcalde et al. (2017) state that a small cell nucleus does not play a crucial role in cell deformability-based experiments under a fluid flow. Therefore, the deformability of the nucleus could be impacted by the size of the nucleus, and thereby influence the penetration time. Whereas, other variables influence the cell penetration time $\tau$ to varying degrees, where the correlation of roughness is the most significant.

To make the problem tractable, some assumptions are made based on the simplified biomedical phenomenon, which are: (1) the equilibrium morphology of the cell is circular in 2D and spherical in 3D, respectively; (2) the cell is not allowed to die, which means the cell cannot be removed, in any extreme narrow scenarios; (3) cell mobility is simulated by a source secreting a single cytokine evenly and continuously until it is consumed , which makes the model consist of a system of ordinary differential equations; (4) the obstacles are absolutely stiff such that they cannot deform and thereby we do not need to consider the degradation of substrate/ ECM on the obstacles. Further, the introduction of elastic obstacles also needs the inclusion of mechanical balance based on Newton’s law for the objects. Although this would be an interesting extension of the model, we omit this in the current paper since this extension enlarges the parameter space for the Monte Carlo simulations. In order to improve the model, the following aspects could be considered in future work.

- Compared to a 2D model, a 3D model is more physiological, however, there is no significant qualitative difference in terms of expected numerical results. Moreover, taking the Monte Carlo simulations into account, the CPU time for simulating the 2D model is much more reasonable. However, a 3D model will still be an interesting research direction in the future.
- Amoeboid and mesenchymal movement, as the two basic forms of cell locomotion, mutually transform and participate in the process of cell migration. The former is also called pseudopodia movement including lamellipodia and filopodia, which normally takes place close to the cell front as a result of cell polarization (Lauffenburger and Horwitz 1996; Lämmermann and Sixt 2009; Paul et al. 2017). Since the interconversion between the amoeboid model and the mesenchymal model due to the cytoskeleton rearrangement happens during cancer cell migration (Zhao et al. 2011), the filopodia that is an extension of active membrane of cell front and rear might be considered in future work.
- In the current work, we define constant values for the cell deformation relaxation $\alpha$ and cell mobility $\beta$ everywhere, while they in general depend on chemokines. Therefore, to introduce surface-resident chemical species, some surface partial differential equations can be incorporated such that it describes the evolution of the chemical signals over the membrane surface. This amounts to solving
  36 $$\begin{aligned} \begin{aligned}&{\underline{a}}_t + \nabla _{\Gamma } \cdot (\mathbf{v} {\underline{a}}) - D_a \Delta _{\Gamma } {\underline{a}} = {\underline{f}} ({\underline{a}}), \\&\mathbf{v} = \frac{\mathrm{d}}{\mathrm{d}t} {\underline{x}}(t), \qquad (t,\mathbf{x}(t)) \in \mathbb {R}^+ \times \Gamma (t). \end{aligned} \end{aligned}$$
  This is an interesting and relevant research direction, which will be taken into consideration in future work.
- A tumor is typically surrounded by a dense network of collagen fibers, which are normally utilized by motile cancer cells to guide their paths (Sahai 2007). Furthermore, mutated cancer cells are capable of remodeling the normal ECM around them, abnormal ECM or the density of fibers preferably reshapes aligned direction in a parallel arrangement, which forms an anisotropic medium and thereby has a significant impact on cell migration. If we formalize this directional dependence through the so-called orientation tensor $\Psi$. Then we get the following revision on the response to the external signal of the migration equations:
  37 $$\begin{aligned} \begin{aligned} \mathrm{d}{} \mathbf{x}_i(t) =\,&(\beta _0 \mathbf{I} + \beta _1 \Psi ) \nabla c(t,\mathbf{x_i}(t)) \mathrm{d}t + \alpha ( \mathbf{x}_i^n(t)\\&+ \hat{\mathbf{x}}_i - \mathbf{x}_i(t)) \mathrm{d}t +\eta \mathrm{d}{} \mathbf{W}(t), \\&i \in \{1,\ldots ,N\}, \end{aligned} \end{aligned}$$
  where $\beta _0$ and $\beta _1$ are two constants and $\Psi$ can be obtained by
  38 $$\begin{aligned} \Psi (t,\mathbf{x}) = \begin{pmatrix} \Psi _{xx} &{} \Psi _{xy} \\ \Psi _{xy} &{} \Psi _{yy} \end{pmatrix}. \end{aligned}$$
  For the formalism, one can refer to the work by Cumming et al. (2010) and a further application in the work (Chen et al. 2018).
- We note that the relaxation parameter of the nucleus has little correlation with the transmigration time. This finding seems counter-intuitive. According to the studies of Serrano-Alcalde et al. (2017), the stiffness of the nucleus hardly plays a role in cell deformability experiments if the nucleus is relatively small. However for larger sizes, this deformability of the nucleus may become more important.

Over the past several decades, a significant progress has been made in medical technology and attempts have been made to investigate the complexity of cancer initiation and progression. For example, cell deformability has been shown to have certain correlations with disease states of cells and metastatic potentials (Guck et al. 2005; Mak and Erickson 2013). Nonetheless, the biological mechanisms of a multi-step metastatic cancer still remain poorly understood (Lambert et al. 2017). To make a contribution, our group will continue to work on biological mathematical modeling to predict the behavior of cells in the microenvironment and aid the biological experiments for the further understanding of cancer and drug development.

## Electronic supplementary material

Below is the link to the electronic supplementary material.

Video1 One cell migrating along a rigid obstacle in 2D simulation. The corresponding consecutive snapshots are shown in Fig.3 of manuscript. (video 2.91 MB)

Video2 One cell migrating along a rigid obstacle in 3D simulation. The corresponding consecutive snapshots are shown in Fig.4 of manuscript. (video 3.65 MB)

Video3 One cell penetrating a cavity made of two rigid obstacles in 2D simulation. The corresponding consecutive snapshots are shown in Fig.5 of manuscript. (video 2.52 MB)

Video4 One cell penetrating through an endothelial cell wall in 2D simulation. The corresponding consecutive snapshots are shown in Fig.6 of manuscript. (video 6.18 MB)

Video5 One cell intravasting and extravasating of a blood or lymphatic vessel in 2D simulation. The corresponding consecutive snapshots are shown in Fig.7 of manuscript. (video 7.55 MB)
